# Differential Expression of Metabolism-Related Genes in Plateau Pika (*Ochotona curzoniae*) at Different Altitudes on the Qinghai–Tibet Plateau

**DOI:** 10.3389/fgene.2021.784811

**Published:** 2022-01-20

**Authors:** Hongjuan Zhu, Liang Zhong, Jing Li, Suqin Wang, Jiapeng Qu

**Affiliations:** ^1^ Key Laboratory of Adaptation and Evolution of Plateau Biota, Northwest Institute of Plateau Biology, Chinese Academy of Sciences, Xining, China; ^2^ University of Chinese Academy of Sciences, Beijing, China; ^3^ Qinghai Province Key Laboratory of Animal Ecological Genomics, Xining, China

**Keywords:** plateau pika, thermogenic capacity, energy metabolism, gene expression, altitude

## Abstract

According to life history theory, animals living in extreme environments have evolved specific behavioral and physiological strategies for survival. However, the genetic mechanisms underpinning these strategies are unclear. As the highest geographical unit on Earth, the Qinghai–Tibet Plateau is characterized by an extreme environment and climate. During long-term evolutionary processes, animals that inhabit the plateau have evolved specialized morphological and physiological traits. The plateau pika (*Ochotona curzoniae*), one of the native small mammals that evolved on the Qinghai–Tibet Plateau, has adapted well to this cold and hypoxic environment. To explore the genetic mechanisms underlying the physiological adaptations of plateau pika to extremely cold ambient temperatures, we measured the differences in resting metabolic rate (RMR) and metabolism-related gene expression in individuals inhabiting three distinct altitudes (i.e., 3,321, 3,663, and 4,194 m). Results showed that the body mass and RMR of plateau pika at high- and medium-altitudes were significantly higher than those at the low-altitude. The expression levels of peroxisome proliferator-activated receptor α (*pparα*), peroxisome proliferator-activated receptor-γ coactivator-1α (*pgc-1α*), and the PR domain-containing 16 (*PRDM16*) in white (WAT) and brown (BAT) adipose tissues of plateau pika from high- and medium-altitudes were significantly higher than in pika from the low-altitude region. The enhanced expression levels of *pgc-1α* and *pparα* genes in the WAT of pika at high-altitude showed that WAT underwent “browning” and increased thermogenic properties. An increase in the expression of uncoupling protein 1 (*UCP1*) in the BAT of pika at high altitude indicated that BAT increased their thermogenic properties. The gene expression levels of *pparα* and *pgc-1α* in skeletal muscles were significantly higher in high-altitude pika. Simultaneously, the expression of the sarcolipin (*SLN*) gene in skeletal muscles significantly increased in high-altitude pika. Our results suggest that plateau pika adapted to an extremely cold environment via browning WAT, thereby activating BAT and enhancing *SLN* expression to increase non-shivering thermogenesis. This study demonstrates that plateau pika can increase thermogenic gene expression and energy metabolism to adapt to the extreme environments on the plateau.

## Introduction

Animals living in different habitats are affected by various ecological factors such as photoperiod, food quantity or quality, and temperature (Van Beest et al., 2012; Hanya and Chapman, 2013; Olanrewaju et al., 2013). To adapt to a changing climate, animals have evolved specialized morphological, behavioral, and physiological traits (Zhu et al., 2017a; Mannuthy, 2017; Fox et al., 2019). For example, an experimental analysis of Trochilidae and *Zonotrichia capensis* showed a correlation between Hb-O_2_ affinity and native elevation (Projecto-Garcia et al., 2013; Cheviron et al., 2014). *Tamiasciurus hudsonicus* and *Lepus americanus* respond to environmental changes by protecting a high and stable body temperature with changes in body temperature and heart rate while reducing behavioral changes (Menzies, 2021). Moreover, as ambient temperature decreases, animals may adjust their behavior and/or physiology to reduce their energy expenditure (Humphries et al., 2005; Zub et al., 2009). Animals such as *Mustela nivalis, Spermophilus parryii*, and *Rhabdomys pumilio* can huddle together or stay in the nest to conserve energy and maintain body temperature (Geiser, 2004; Scantlebury et al., 2006; Sukhchuluun et al., 2018). Many studies have shown phenotypic and physiological adaptations to the environment, and that species-specific adaptations to extreme environments are reflected at the gene transcription level. Studies on *Anolis carolinensis*, *Rhinopithecus bieti*, *Thermophis baileyi*, and *Sus scrofa* have uncovered the gene-expression mechanisms underlying their behavioral and physiological adaptations (Li et al., 2013; Yu et al., 2016; Li T. et al., 2018; Kabelik et al., 2021).

Energy metabolism plays an important role in physiological adaptation, which influences animal distribution, abundance, reproductive success, and fitness (Yaskin, 2011; Healy et al., 2013; Tickle et al., 2018). Energy metabolism is affected by environmental and physiological factors, including body mass, food quality/quantity, and temperature, which substantially affect an animal’s heat production and thermoregulation (McNab, 2009; Tattersall et al., 2012). Elevated thermogenic capacity is crucial to an animal’s survival in a cold environment (Zhang et al., 2017). Thermogenic capacity can be measured as maximum metabolic rate, which is comprised of resting metabolic rate (RMR), shivering thermogenesis (ST), and non-shivering thermogenesis (NST) (Nespolo et al., 2001; Chi and Wang, 2011; Mineo et al., 2012). Compared with those species inhabiting cold environments, animals inhabiting warm environments, i.e., *Meriones unguiculatus* and *Diplolaemus leopardinus*, have a lower RMR (Ding et al., 2018; Vicenzi et al., 2021). Similarly, the RMRs of *Tupaia belangeri* and *Chaetops frenatus* in winter are usually higher than in summer (Zhu et al., 2012; Oswald et al., 2018). Furthermore, animals can adapt to the ambient temperature by changing their thermogenic characteristics, such as increasing protein content, cytochrome *c* oxidase activity, and leptin expression (Meyer et al., 2010; Wang et al., 2019).

As the highest plateau on Earth, the Qinghai–Tibet Plateau has an average altitude of more than 4,000 m. Its unique topography has formed extreme environmental and climatic characteristics (Sun et al., 2014). Animals that inhabit the plateau at high altitudes face the challenging environment of hypoxia and low ambient temperatures (Wang et al., 2011). The Qinghai–Tibet Plateau is one of the most sensitive regions to global climate change (Liu and Chen, 2000). A progressive reduction in temperature occurs with the ascent to high elevation, and high-altitude environments mean considerable physiological challenges to animals (Storz and Scott, 2019). Animals may adjust their physiological characteristics by spending energy to generate heat to survive in high-altitude environments. One important question is how animals regulate their metabolism and maintain their effective energy in extreme environments (O’Brien et al., 2020). Studies of passerine birds, lizards, and *Parnassius* butterflies inhabiting the three high-altitude regions of the Qinghai–Tibet Plateau found that their gene expression correlates with altitude, suggesting that high-altitude environments may drive similar expression patterns in high-altitude species (Yang et al., 2015; Hao et al., 2019; Su et al., 2020).

Adipose tissue, which can be divided into white adipose tissue (WAT) and brown adipose tissue (BAT) in mammals, plays an extremely important role in the regulation of energy homeostasis in animals (Harms and Seale, 2013; Elsen et al., 2014). The PR domain of 16 (*PRDM16*) and peroxisome proliferator-activated receptor γ coactivator-1α (*pgc-1α*) were key transcriptional regulators in mice and induced classic brown fat accumulation in hypothermia induction (Seale et al., 2011). Peroxisome proliferation receptor-α (*pparα*) mediated lipid thermogenesis by sensing *pgc-1α* and *PRDM16* expression as a key component of brown fat thermogenesis (Hondares et al., 2011). Studies have shown that the deletion of the *SLN* gene in skeletal muscle causes mice to fail to maintain body temperature during exposure to acute cold, demonstrating that sarcolipin (*SLN*) is an important player in adaptive thermogenesis (Bal et al., 2012). There was also an increase in the transcriptional regulators of mitochondrial biogenesis, such as *pparα* and *pgc-1α* (Handschin et al., 2003; Ryder et al., 2003; Schaeffer et al., 2004). The metabolic function of the liver is controlled by insulin and other metabolic hormones. Studies have shown that under food serious shortage, cAMP-response element binding protein (CREB) and *pgc-1α* are key transcriptional coactivators in hepatic gluconeogenesis in two experimental mouse models; they play a key role in maintaining long-term gluconeogenesis (Herzig et al., 2001; Oh et al., 2013).

The plateau pika (*Ochotona curzoniae*) is a keystone species on the Qinghai–Tibet plateau (Yu et al., 2012) and plays an important role in maintaining the biodiversity and stability of the alpine meadow ecosystem (Smith and Foggin, 1999; Wilson and Smith, 2015). It inhabits the alpine regions at an altitude of 3,100–5,300 m above sea level and is well adapted to extreme hypoxia, cold, and food deprived environments (Cao et al., 2017). In this scenario, plateau animals including plateau pika face severe energetic challenges to maintain their core body temperature (Van Sant and Hammond, 2008; Zhang et al., 2012; Speakman et al., 2021). Previous studies have found that at different altitudes, the life history strategies and personalities of plateau pika varied significantly (Liu et al., 2012; Qu et al., 2013; Qu et al., 2019; Tan et al., 2020), accompanied by differences in fat accumulation and metabolic rate (Yang et al., 2006). As ambient temperatures decrease, subcutaneous WAT “browned”, and adipose tissue heat production increased (Bai et al., 2015; Li et al., 2019). However, studies on the expression of thermogenic genes in adipose tissue and other thermogenic tissues of plateau pika at different altitudes are limited. In the current study, we live-trapped plateau pika at different altitudes. We measured their metabolic rate and transcriptome expression levels in adipose tissue, liver, and skeletal muscle in order to profile gene expression patterns and investigate the role of transcriptional regulation in tissue-level metabolic adaptation to high altitudes. We aimed to test the following hypotheses: 1) the RMR of the plateau pika increases with rising altitude, and 2) metabolism-related gene expression synchronously increases with rising altitude, adapting to the extreme environments of the Qinghai–Tibet Plateau.

## Materials and Methods

### Animals and Sample Collection

Plateau pikas inhabiting high-, middle-, and low-altitude regions were live trapped from Maduo, (4,194 m above sea level, *n* = 24), Guide, (3,663 m, *n* = 24), and Guinan (3,321 m, *n* = 24), respectively, in Qinghai Province, in December 2020. Maduo has an annual average temperature of −4°C and an average monthly temperature below −3.0°C, classifying it as an alpine steppe climate. The annual average temperature of Guide is −3.7°C; a plateau continental climate. Guinan does not experience a severely cold winter or an intensely hot summer, and the annual average temperature is 2.3°C; as such, it is also considered a plateau continental climate.

Ten pikas from each altitudinal region were immediately anesthetized and dissected after capture (five females and five males at each altitude). The adipose tissue, liver, and muscle tissue were immediately preserved in liquid nitrogen and stored at −80°C until further RNA extraction and analyses. A further sample of plateau pikas (*n* = 14) from each altitude were live-transported to the animal laboratory in Xining (2,261 m above sea level, outdoor temperature −2°C, indoor temperature 20°C). They were kept in 545 × 395 × 200 mm polypropylene material cages separately under 12 L: 12 D lighting conditions, and provided with artificial food (Tianjin Tongyu Feed Sales Co. Ltd.) and ad libitum water. Metabolic experiments were conducted within 24 h.

### Metabolic Trials and Non-shivering Thermogenesis (NST)

The RMR of plateau pikas were expressed as oxygen consumption per hour per unit body mass [mL O_2_/(g·h)] and measured using an 8-channel FMS (Sable Systems International, Henderson, NV, United States) portable respiratory metabolism system. A biochemical incubator was used to control the chamber temperature, and the experimental temperature was set at 27.5°C (which is within the pika thermal neutral zone) with a standard error of 0.5°C. Metabolic measurements were conducted after the pikas had acclimatized in the chamber for 0.5 h and were resting. The RMRs of seven pikas were measured simultaneously, and a blank tube was used as the baseline for carbon dioxide, oxygen, water vapor, and temperature (Tan et al., 2020; Yu et al., 2021). Four rounds of metabolism were measured in 2 h, with each round lasting 30 min. When the chamber temperature was 27.5°C, the average of the lowest metabolic rates of each individual over at least 10 min was selected as the RMR (Boratyński et al., 2017). RMR is the minimum energy requirement for animals to maintain normal physiological activities within a thermally neutral environmental temperature, while at rest (Arnold et al., 2021). Before the experiment, the pikas were fasted for 2–3 h, and their body mass and temperature were measured using an electronic balance and rectal thermometer, respectively. A digital thermometer probe was inserted gently about 2 cm into the rectum; the measurement time did not exceed 30 s.

Noradrenaline (NE) induction is widely used to determine NST because induced heat generation and cold induction are equivalent and the mechanism is the same. The dose was 0.7 mg/kg in reference to the seasonal variation of NST in plateau pika measured by Wang (Wang and wnag, 1990). The 10 plateau pikas at each altitude were brought back to the laboratory and allowed to adapt for 24 h. The pikas were raised in a single cage under 12 L:12 D illumination in the laboratory, fed with sufficient amounts of rabbit pellet feed (Jiangsu Syu Pharmaceutical Biological Engineering Co., Ltd.), provided water ad libitum, and adapted for 2–3 h before the experiment. NST was measured using an 8-channel FMS respiratory metabolic measurement system. NE was injected subcutaneously into the back with a dose equivalent to pika body weight (0.4 mg/kg). The pikas were immediately put back into the respiratory chamber for 30 min. NE was injected with norepinephrine (1 ml containing 2 mg), having been diluted to 0.4 mg/ml by adding normal saline. The NE was produced by Shanghai Wellhope Pharmaceutical Co., Ltd. In general, the peak in metabolic response occurs 10–45 min after the NE injection. A scatterplot of oxygen consumption against determination time was generated, and the average value of 10 consecutive and stable maximum values was taken as the NST value.

### Reverse Transcription (RT) and Quantitative Real-Time PCR (qPCR)

qRT-PCR was used to determine the expression of *pparα*, *pgc-1α*, CREB, *PRDM16*, *SLN* and uncoupling protein 1 (*UCP1*). The species-specific primer sets and 18s-actin of the genes in plateau pika were designed in accordance with the reference gene sequences in the NCBI (national center for biotechnology information) website (http://www.ncbi.nlm.nih.gov/) for reference North American pika gene sequences. Primer6.0 software was used to design primers.

Total RNA was extracted from tissues using the Uniq-10 Column Trizol Total RNA Extraction Kit (B511321) in accordance with the kit instructions. The primer was set for 18s and six transforming genes were designed for quantitative real-time PCR. Quantitative real-time PCR was completed using the 2SG Fast qPCR Master Mix (B639271, BBI, Roche) in the LightCycler480 II type fluorescent quantitative PCR instrument (Roche, Rotkreuz, Switzerland). qRT-PCR was carried out in a 10-μL reaction system, which was composed of 5 μL of 2 SybrGreen qPCR Master Mix, 1 μL of cDNA and 0.2 μL of each primer (10 μM/L), and 3.6 μL of ddH_2_O. All PCR reactions were repeated. The thermal cycling conditions were as follows: 95°C for 3 min, 45 cycles at 95°C for 5 s, and 60°C for 30 s. The melting curve analysis revealed genes and 18s-amplified single-PCR and final products. We constructed a standard curve for each gene by diluting the cDNA sequence fivefold. The standard curve analysis of target genes and 18 s showed that they had similar amplification efficiency, which ensured the effectiveness of the comparative quantification method. Gene expression was calculated using the 2^−ΔΔCt^ method and expressed as relative quantities. The nucleotide sequences of primers used for qPCR are shown in Supplementary Table S1.

### Statistical Analysis

All data analyses were conducted using R 3.4.3 software. Body mass, RMR, and metabolism-related gene expression levels were analyzed using two-way analysis of variance. Prior to all statistical analyses, data were examined for assumptions of normality and homogeneity of variance by using Shapiro–Wilk and Levene tests, respectively. Differences among groups were detected using Duncan’s multiple range test. Results were presented as mean ± 0.5 standard error (SE); n is the sample size. *p* < 0.05 was considered statistically significant.

## Results

### RMR and Body Mass

**FIGURE 1:**
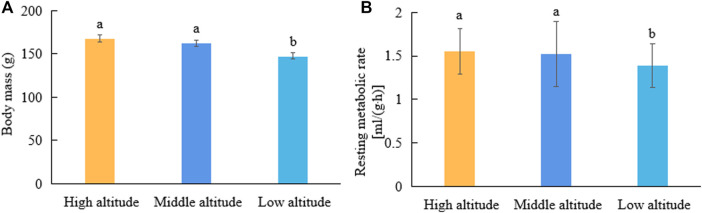
Comparisons of body mass and RMR of plateau pikas from regions with different altitudes. Different lowercase letters indicate significant differences among elevations (*p* < 0.05). Same lowercase letters indicate have not significant difference among elevations (ns *p* > 0.05).

The body masses of plateau pikas from the high- and middle-altitude regions were significantly higher than those from low-altitude regions (F = 7.16, *p* < 0.05; Figure 1A). The mass-corrected RMRs of plateau pikas were 1.55 ± 0.18, 1.52 ± 0.33, and 1.39 ± 0.17 ml/(g·h) in high-, middle-, and low-altitude regions, respectively. The RMRs of plateau pikas from high- and middle-altitude regions were significantly higher than those from the low-altitude region (F = 3.49, *p* < 0.05; Figure 1B).

### Gene Expression in Adipose Tissue

**FIGURE 2:**
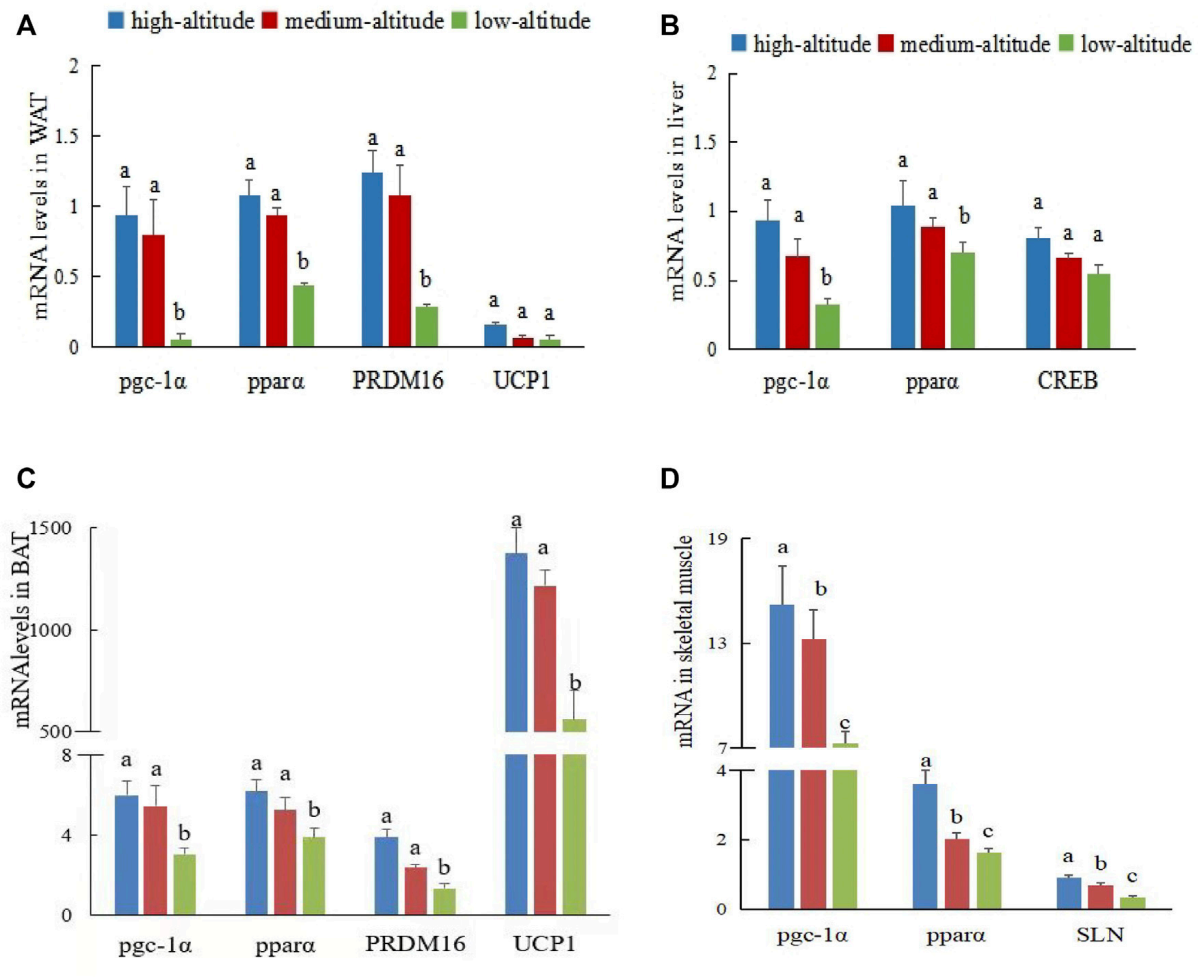
Gene expression of different tissues in plateau pika from regions with different altitudes. Note: **(A)** Expression of *pgc-1α*, *pparα*,*PRDM16* and UCP1 genes in WAT. **(B)** Expression of *pgc-1α*, *pparα* and *CREB* genes in liver. **(C)** Expression of *pgc-1α*, *pparα*, *PRDM16*, and *UCP1* genes in BAT. **(D)** Expression of *pgc-1α*, *pparα*, and *SLN* genes in skeletal muscle. Different lowercase letters indicate significant differences among elevations (*p* < 0.05). Same lowercase letters indicate have not significant difference among elevations (ns *p* > 0.05).

To explore molecular signatures of the thermogenesis of WAT and BAT, we performed profiling of gene expression in the two fat tissues from the three elevation groups. No significant difference was observed in gene expression between the sexes (*F* = 0.36, *p >* 0.05), whereas significant differences were detected between the three altitudes (*F* = 15.56, *p <* 0.05). *UCP1*, *PRDM16*, and *PGC-1a* are the key transcriptional regulators associated with browning and BAT, they were the transcriptional co-activator that is involved in browning and mitochondrial biogenesis. The expression levels of the *UCP1* protein in BAT from high- and medium-altitude regions were higher than those from the low-altitude region, which indicates that BAT is specialized for NST and energy dissipation through the action of *UCP1*. Evidence for an increase in NST is provided in the supplementary material (Supplementary Figure S1). Similar results were obtained for *pgc-1α* (Figures 2A,C). The expression levels of *pgc-1α*, *pparα*, and *PRDM16* genes in the WAT and BAT of plateau pikas from high- and medium-altitude regions were significantly higher than those from the low-altitude region (*p <* 0.05; Figures 2A,C).

### Gene Expression in Liver

Liver is a metabolic organ, and its metabolic function is controlled by metabolic hormones such as insulin. To explore the molecular characteristics of hepatic gluconeogenesis in liver, we detected the differential expression of energy metabolism molecules in three plateau pika samples at different altitudes. No significant difference was observed between the sexes (*F* = 1.587, *p >* 0.05), whereas a significant difference in gene expression levels was found between altitudes (*F* = 13.59, *p <* 0.05). The main transcription factors inducing gluconeogenesis include CREB, FoxO1, and several nuclear receptors. *PGC-1α* is a key transcriptional coactivator for FoxO1 in hepatic gluconeogenesis, which plays a key role in maintaining long-term gluconeogenesis under conditions of scarce food resource. The expression levels of *pgc-1α* and *pparα* genes in the liver of plateau pikas from the high-and medium-altitude regions were significantly higher than those from the low-altitude region, but the expression levels of the CREB gene were not significantly different between the three regions (*p >* 0.05, Figure 2B).

### Gene Expression in the Skeletal Muscle

As the largest organ, skeletal muscle is also a major contributor to metabolic rate and can significantly affect metabolism and body weight by increasing muscle energy expenditure through non-shivering thermogenesis. We studied the differential expression of thermogenic molecules in the skeletal muscle of pikas at the three altitudes. No significant difference was observed in gene expression between the sexes (*F* = 0.78, *p >* 0.05), but gene expressions in the three regions were significantly different (*F* = 11.56, *p <* 0.05). The expression levels of *pgc-1α*, *pparα*, and *SLN* genes in the muscle of plateau pikas from the high-altitude region were significantly higher than those from the medium-altitude region (*F* = 15.49, *p <* 0.05). The expression levels of *pgc-1α*, *pparα*, and *SLN* genes of plateau pikas from the medium-altitude region were significantly higher than those from the low-altitude region (*F* = 14.91, *p* < 0.05; Figure 2D).

## Discussion

Adaptive evolution is a hot topic in evolutionary ecology. Elucidating the selection pressures that drive the evolution of metabolic rate is fundamental to understanding the evolution of the morphology, physiology, behavior, and life histories of animals (McKechnie and Swanson, 2010). In the present study, the metabolic rates of plateau pikas from the high- and middle-altitude regions were significantly higher than those from the low-altitude region. The expression levels of *pparα*, *PRDM16*, and *UCP1* in the WAT and BAT of plateau pikas from high and medium altitudes are significantly higher than in those from low altitude. Simultaneously, the expression levels of *SLN* genes in skeletal muscle and liver significantly increase in high-altitude pikas. These data support the contention that through long-term adaptation, the plateau pika has adapted to high altitude and evolved efficient approaches to deal with the extreme cold and harsh environments on the Qinghai–Tibet Plateau.

### RMR and Body Mass

Selective pressures affecting metabolism are complex and can influence metabolic rate through multiple pathways (Zheng et al., 2014b) such as body size, climate, activity, and habits (Killen et al., 2016). Body mass is the most direct indicator of animal energy reserves (Swanson et al., 2017). For example, statistical tests found that the metabolic rate of 533 species of birds was positively correlated with body mass (McNab, 2009). In the present study, the body masses of plateau pikas from high- and middle-altitudes were significantly higher than those from the low altitude. Significant correlations between body mass and metabolic rate were found in the hamster subfamily (Bozinovic, 1992). According to Bergmann’s law, the increase in body mass decreases the surface-to-volume ratio, thereby reducing heat loss and living costs (Zheng et al., 2014a). Many variables associated with physiology are correlated with latitude, indicating that climate is an important factor for the evolution of life-history traits (Tószögyová, 2020). Meta-analyses about the metabolic rate of 69 species of tropical birds and 59 species of temperate birds found that tropical migrants in temperate habitats have lower metabolic rates than do temperate residents (Wiersma et al., 2007). Compared with *Cricetulus barabensis* kept at room temperature, the energy intake of individuals adapted to a low temperature was higher while the energy intake of individuals adapted to a high temperature was lower (Zhou et al., 2015). The RMRs of plateau pikas in the current study inhabiting high altitude are significantly higher than those of pikas inhabiting low altitude. High metabolic rates may be caused by the biochemical activities of several tissues including the liver, BAT, and skeletal muscle, which all have high mitochondrial oxidative phosphorylation rates (Burton et al., 2011; Selman et al., 2013). Increased metabolism plays an important role in thermal regulation in animals living in an extremely cold environment (Gordon, 2012; McKie et al., 2019).

### Gene Expression in WAT and BAT

**FIGURE 3:**
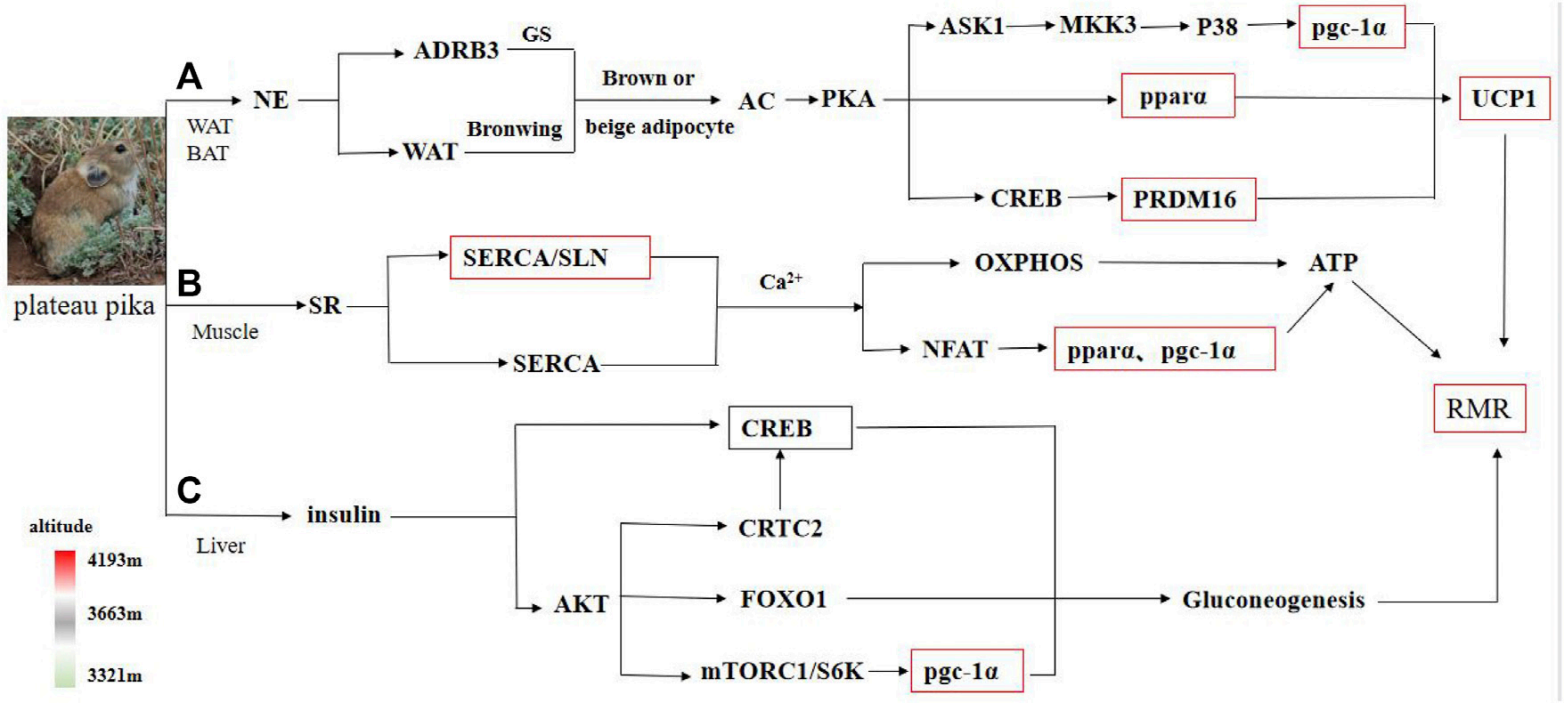
Schematic of energy metabolism in various tissues of plateau pika. Note: The red box represents significant change in gene expression, whereas the green box represents no significant change in gene expression among regions with different altitudes. **(A–C)** represent the metabolic process of adipose tissue, muscle tissue and liver tissue, respectively.

Cold and hypoxia are defining features of the Qinghai–Tibet Plateau environment, and plateau pika have developed tolerances to this harsh environment (Xie et al., 2014; Wei et al., 2016). Consistent with previous studies that demonstrated that pika have tolerance to hypoxia and low-temperatures (Yang et al., 2006; Zhu et al., 2018), a previous study showed that plateau pika can effectively endure extremely cold environments (Li et al., 2001). Earlier studies found that plateau pika have high NST to cope with the cold environment on the plateau in comparison to Ochotonidae from other regions (Wang et al., 2006; Luo et al., 2008). NST is related to tissue heat production, especially adipose tissue, which is important in regulating body temperature and energy homeostasis in cold environments (Zhu et al., 2017b). As two major types of adipose tissue, WAT is involved in energy storage and BAT is involved in energy expenditure and thermogenesis. BAT and WAT can be conditionally interconverted in response to neuroendocrinal factors, β-3-adrenergic stimulation, and cold stress exposure. WAT responds quickly to environmental changes under cold conditions and takes on the characteristics of BAT. When animals inhabit a cold environment, WAT may possibly transform into beige and brown adipocytes to increase NST in order to adapt to cold conditions (Nedergaard et al., 2007) (Figure 3A).

The intermittent cold exposure experiment demonstrated that plateau pika kept in warm temperatures have little classical brown fat, but the “browning” of WATs is detected rapidly upon cold exposure. The expression of several brown fat differentiation markers, including *UCP1*, increases simultaneously. The increase in *UCP1* expression enhances adaptive thermogenesis (Bai et al., 2015). The study about *Tupaia belangeri* shows that RMR and the expression levels of *pparα*, *pgc-1α*, and *PRDM16* increase significantly under cold acclimation, suggesting that browning may appear in WAT (Hou et al., 2020). A genomic, proteomic, and morphological study of energy metabolism in highland pikas and *Tupaia belangeri* in summer and winter studies revealed that subcutaneous WAT in winter show BAT morphological and histological features (Li et al., 2019). Furthermore, BAT-specific genes, such as *UCP1*, *Cox4*, and *pgc-1α*, are highly expressed in WAT in winter (Li J. et al., 2018). These results suggest that plateau pika adapt to a cold environment by browning scarfskin WAT and adding BAT to increase heat production. Our results suggest that a high expression of *pgc-1α* may be involved in the critical adaptation mechanisms in pika to cope with the harsh environment of the Qinghai–Tibet plateau. *pgc-1α* is essential for brown fat thermogenesis and complementary mitochondrial biogenesis, and is also involved in the browning of WAT (Finck and Kelly, 2006). High mRNA expression levels of *pgc-1α* are observed in high-altitude groups, suggesting high levels of thermogenesis within the tissues. Overall, our study indicated that plateau pikas inhabiting the high altitudes of the Qinghai–Tibet Plateau can regulate their relative gene expression in adipose tissue to, in turn, regulate metabolic level and thermogenic-related physiological performance.

### Gene Expression in Skeletal Muscle

The skeletal muscle is a major determinant of basal metabolic rate (Maurya et al., 2018). Skeletal muscle also plays a central role in temperature homeostasis and can be recruited to produce heat through NST (Nowack et al., 2017). As an uncoupler of the sarcoplasmic reticulum calcium ATPase (SERCA) pump, *SLN* can enhance futile cycling and increase ATP hydrolysis, thereby creating chronic energy demand (Sahoo et al., 2013). The *SLN/SERCA* interaction plays a dual role: it creates energy demands in muscle and activates Ca^2+^-dependent signaling, such as *pgc-1α* and *pparα*, to increase ATP production through increased mitochondrial biogenesis (Shaikh et al., 2016) (Figure 3B). In genetically engineered *SLN* mouse models, *SLN* knockout mice have reduced cold adaptive thermogenesis (Bal et al., 2018). The loss of *SLN* predisposes mice to diet-induced obesity, indicating that *SLN* may regulate their energy balance (Bal et al., 2012). Compared to *SLN* gene-lacking mice, the overexpression of the *SLN* gene leads to a loss of body mass and increases in the depletion of fat deposits (Rotter et al., 2018). The Rotter et al. (2018) study was conducted at thermoneutrality, which can minimize the contribution to metabolic rate of thermogenic mechanisms. Thus, a high energy consumption may be due to *SLN*-mediated energy expenditure (Maurya et al., 2015). In the present study, the *SLN* gene expression level of plateau pika from the high-altitude region was significantly higher than that of plateau pika from the middle-altitude region, which in turn was significantly higher than the *SLN* gene expression of pika from the low-altitude region. This result suggests that the *SLN* gene is important in regulating the heat production of plateau pikas at different altitudes.

### Gene Expression of Liver

The liver is an essential metabolic organ, and its metabolic function is regulated by insulin and other metabolic hormones. Numerous transcription factors and coactivators, including CREB, pparg, and pgc-1 regulate the expression of enzymes that catalyze key steps of metabolic pathways, thus managing the energy metabolism of liver (Rui, 2014). When food is scarce, the hepatic gluconeogenesis pathway is enhanced by decreasing the concentration of insulin and increasing the concentration of insulin counter-regulatory hormones, such as glucagon (Han et al., 2016). *pgc-1α*, CREB/CRTC2, and FoxO1 genes are critical in coordinating the fasting-mediated activation of gluconeogenesis in the liver (Oh et al., 2013) (Figure 3C). In our study, the *pgc-1α* and *pparα* in the liver of plateau pika from the high-altitude region were significantly higher than those of pika from the low-altitude region, whereas no significant difference in the CREB gene was detected among the three regions, suggesting that ATP depletion is due to activity-induced energy demands and the storage of fatty acids, cholesterol, glycogen, and proteins, especially in liver (Ke et al., 2018).

In conclusion, the RMR, and the expression of skeletal muscle thermogenic genes and lipid transcription factor genes in plateau pika increases with rising altitude on the Qinghai–Tibet Plateau. Therefore, plateau pikas inhabiting high-altitude environments can survive extreme environments by increasing their metabolic rate, and gene expression of skeletal muscle thermogenesis and adipose tissue. Browning increases the expression of *UCPI* to promote BAT cell differentiation, thermogenesis, and metabolism. These physiological and gene expression changes confer plateau pika the ability to survive in an extreme environment.

## Data Availability

The datasets presented in this study can be found in online repositories. The names of the repository/repositories and accession number(s) can be found in the article/Supplementary Material.
