# Supplementary material for: Differential Expression of Metabolism-Related Genes in Plateau Pika (Ochotona curzoniae) at Different Altitudes on the Qinghai–Tibet Plateau
Source: Front Genet. 2022 Jan 20;12:784811. doi: 10.3389/fgene.2021.784811 (PMC8811202; doi:10.3389/fgene.2021.784811)
Supplement: Supplementary file 2 [file DataSheet1.docx]

Supplementary Materials

Table 1. RT-PCR primer sequences

| Name | Sequence (5′–3′) |
| --- | --- |
| 18sRNA | 18sRNA-F AGGCCATGATTAAGAGGGAC |
|  | 18sRNA-R TCTGATCGTCGTCGAACCTC |
| *pgc-1α* | pgc-1α-F AGGGCGAAACAAAGGGAGA |
|  | pgc-1α-R CGTCACAGGTGTATCGGTAGGT |
| *pparα* | pparα-F ACCTGGAAAGCCCGTTATCT |
|  | pparα-R ACCACAGGACAAGTGACGGAT |
| *PRDM16* | PRDM16-F3 TCCTACACGCAGTTCTCCAAC |
|  | PRDM16-R3 GTAATGGTTCTTGCCCTCGC |
| UCP1 | UCP1-F GGGAGGAAGCAACACCAAGT |
|  | UCP1-R CCGTGTAGCGAGGTTTGAGA |
| CREB | CREB-F CTGGACCCTGAAGAGACGACT |
|  | CREB-R CTGGAGGTTGGGATTGCTTA |
| SLN | SLN-Fn ACGCACAATGTTGACATACTCC |
|  | SLN-Rn AACCCTGAGGCTTGTCTAATGT |

Figure S1 Comparisons of nonshivering thermogenesis of plateau pikas from regions with different altitudes.

Figure S2 Comparison of resting metabolic rate of plateau pikas at different altitudes at ambient temperature of 15℃.
